# Japan’s Four-Decade Natural Experiment in Early Childhood Caries: A Perspective on Prevention Pathways Beyond Fluoride

**DOI:** 10.1016/j.identj.2026.109473

**Published:** 2026-02-28

**Authors:** Yoshihisa Yamashita

**Affiliations:** Division of Infectious and Molecular Biology, Kyushu Dental University, Kitakyushu, Japan

**Keywords:** Early childhood caries, Fluoride toothpaste, Japan, Maternal–child health, Natural social experiment, Caries epidemiology

## Abstract

Japan’s marked decline in early childhood caries (ECC) has been documented through uniquely comprehensive national dental examinations, with standardized population surveys conducted continuously for more than four decades. This perspective discusses how Japan achieved sustained reduction in ECC despite the absence of community water fluoridation and historically limited use of toothpaste with effective fluoride concentration in infancy. Drawing on national surveillance and epidemiological evidence, I consider how changes in dietary patterns, caregiver practices, oral health behaviours, and broader social conditions may have contributed to a less cariogenic environment for young children. Importantly, this experience does not diminish the established effectiveness of fluoride-based interventions; rather, it highlights complementary, system-level factors operating over long time horizons. Japan’s experience may inform context-sensitive ECC prevention strategies, particularly in settings where fluoride use is limited, unevenly implemented, or difficult to sustain.

## Introduction

Early childhood caries (ECC) remains a major global health problem affecting hundreds of millions of children worldwide.^1^, ^2^, ^3^, ^4^ Its aetiology is multifactorial, shaped by biological, behavioural, social, and structural determinants. Fluoride plays a central role in caries prevention across the life course; however, evidence supporting its use before age three remains limited and inconsistent. International guidelines^5^^,^^6^ recommend 1000 ppm fluoride toothpaste from the eruption of the first tooth, but the underlying evidence is weak, and risk–benefit considerations vary substantially across contexts.

Japan presents a uniquely informative epidemiological model. Despite minimal fluoride exposure in infancy – no community water fluoridation, and the prevailing professional recommendation for low-fluoride toothpaste (500 ppmF) until 2023 – ECC prevalence in 3-year-olds has declined continuously for more than four decades.^7^^,^^8^ Given Japan’s near-universal maternal–child health (MCH) infrastructure and historically high attendance at 3-year-old dental checkups, this long-term decline provides a rare opportunity to examine ECC reduction under conditions where fluoride exposure played only a minor role.

**Fig. 1:**
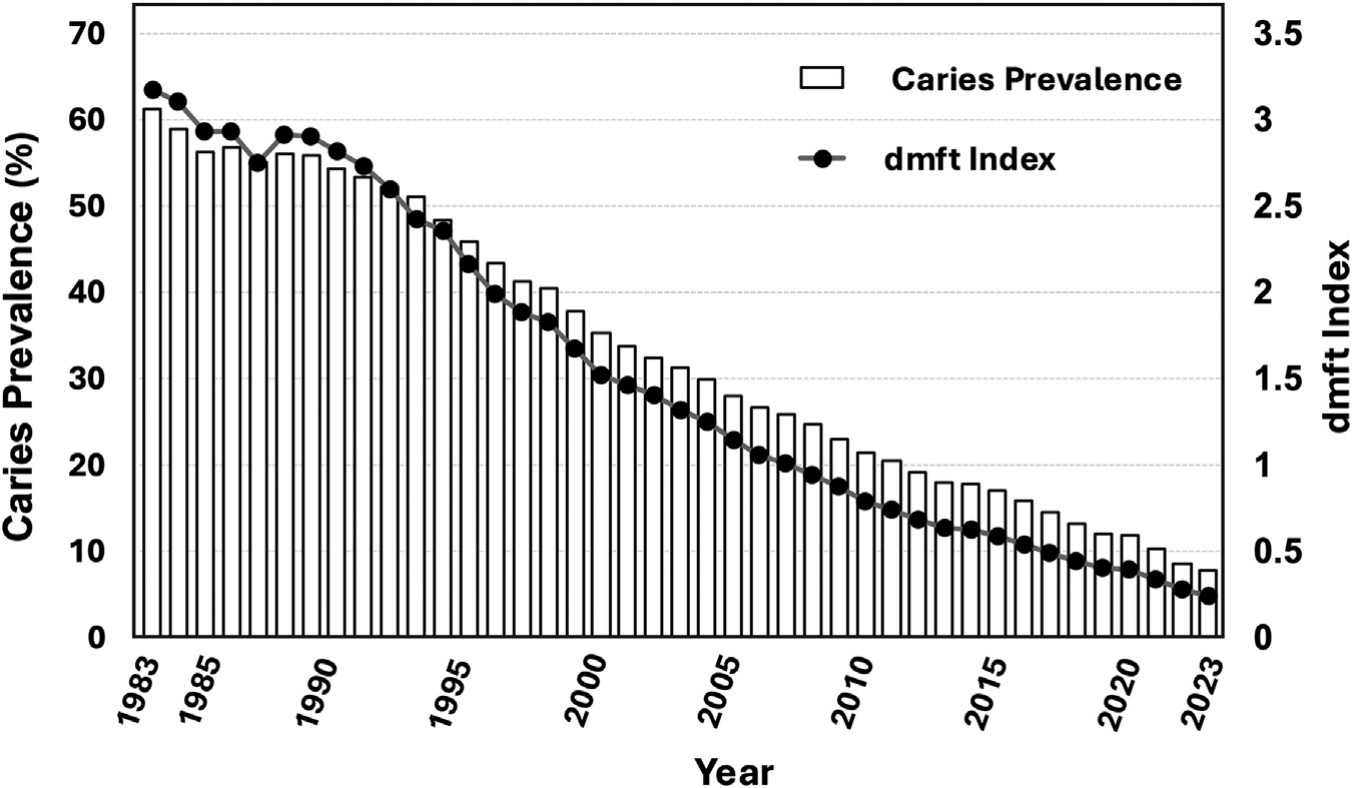
Trends in dental caries among 3-y-old children in Japan (1983-2023). Caries prevalence showed a continuous decline over the four decades, accompanied by parallel reductions in mean dmft (representing the average number of decayed, missing, and filled primary teeth per child). Together, these trends indicate substantial reductions in both the proportion of affected children and the severity of disease among those with caries. Data from nationwide municipal dental checkups.

Understanding the determinants of Japan’s remarkable ECC decline (as illustrated in Figure 1) may help refine the evidence base for early-life caries prevention and inform global policy, particularly in settings where early-life caries risk is already low.

## Japan’s 3-year-old dental checkup system

Japan’s MCH program is universal, longitudinal, and municipally administered. The 3-year-old dental checkup became legally mandated for municipalities in 1994, with full organizational transition completed by 1997.^7^^,^^8^ These examinations are free of charge, standardized, and embedded within a broader series of developmental checkups conducted at 1.5 and 3 years of age.

**Fig. 2:**
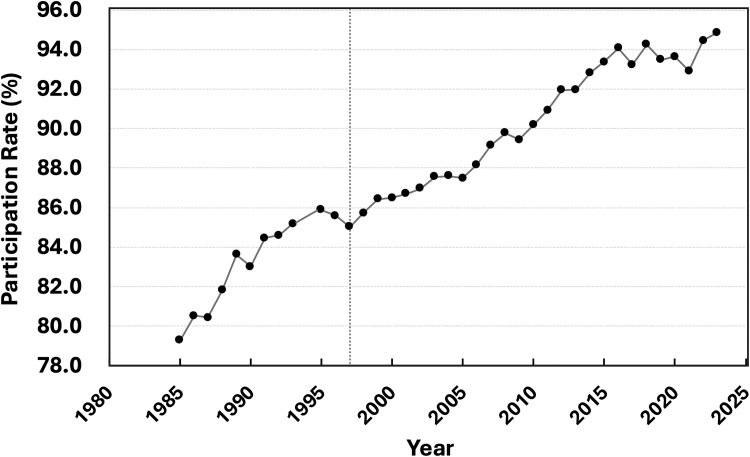
Participation rates in Japan’s nationwide mandatory 3-y-old dental checkups (1985-2023). Participation rates remained stable or increased over time, recently reaching approximately 95%, making it unlikely that long-term national ECC trends reflect selection bias. The vertical dashed line marks 1997, the year when administrative responsibility for the 3-y-old dental checkups fully transitioned to municipalities under Japan’s maternal–child health system. Data from nationwide municipal dental checkups.

Because the checkups are mandatory for municipalities and financially accessible to all families, participation remains consistently high. Figure 2 shows that attendance has remained stable or increased over time, with participation rates exceeding 90% since 2010 and reaching approximately 95% in recent years. Such stable or increasing attendance is unlikely to account for the observed long-term decline in ECC. Moreover, higher attendance does not necessarily imply lower caries prevalence, as increased participation may preferentially include children from higher-risk families seeking consultation or treatment. Therefore, stable or increasing attendance alone cannot explain the sustained, near-linear decline observed over four decades. Very few countries have oral health datasets of comparable completeness, duration, and methodological consistency.^7^^,^^9^^,^^10^

Although the data originate from routine public health services rather than research-specific surveillance, the diagnostic results have shown exceptional year-to-year stability. Over four decades, ECC prevalence at both the 1.5- and 3-year-old examinations has declined along a nearly linear trajectory with minimal annual fluctuation. Such consistency is rarely seen in large-scale, non-research clinical datasets and likely reflects the high degree of standardization in examination procedures, examiner training, and municipal implementation. The smooth, continuous downward trend provides strong evidence that these data capture true population-level changes rather than measurement artefacts and are in many respects more stable than typical sampling-based research datasets.

This highly reliable system of municipal dental checkups underpins all longitudinal ECC trends described in this Perspective.

## Long-term trends in ECC in Japan

Mean dmft scores declined consistently alongside reductions in untreated caries. Although individual trends in treated and untreated components were not separately analysed in this Perspective, the steady decline in dmft, together with the marked long-term reduction in overall caries prevalence and the corresponding increase in the proportion of caries-free children, indicates that the overall burden of disease genuinely decreased and is unlikely to be explained solely by changes in treatment patterns.

These patterns are particularly notable given Japan’s minimal fluoride exposure in early childhood during these years. Until 2023, the fluoride concentration of children’s toothpaste recommended by Japanese dental societies was 500 ppmF – a level considered insufficient for caries prevention by WHO and FDI guidelines.^6^^,^^11^ Japan has never adopted community water fluoridation, and overall fluoride exposure in infancy remained low throughout the entire period of decline.

Because fluoride-based explanations are insufficient to account for this decades-long decline, attention must shift to alternative contributors. Multiple non-fluoride factors likely interacted over time to produce the observed improvements, including behavioural, social, dietary, and structural determinants. These potential mechanisms are explored in detail in Section 4.

## Potential mechanisms behind Japan’s ECC decline

### Behavioural improvements

Parental oral-hygiene practices appear to have improved over several decades, with earlier initiation of toothbrushing, more regular brushing routines, and increasing access to age-appropriate guidance through Japan’s MCH system. National oral health surveys, including the Dental Diseases Survey,^12^ have documented long-term increases in daily toothbrushing frequency among adults and caregivers. Broader improvements in oral-hygiene awareness at the population level have also been reported.^8^ Although these surveys do not directly assess hygiene practices among children under three, such population-wide improvements in caregiver behaviours likely enhanced the early home oral-health environment in which young children are raised.

### Dietary and lifestyle changes

Japan experienced a substantial and prolonged reduction in per-capita sugar consumption over the past 5 decades. National food-supply statistics show that annual sugar intake decreased substantially from the early 1980s to about 14 to 15 kg per person in 2023, representing a reduction of approximately 40% to 45%.^13^ Japan now has one of the lowest per-capita sugar-consumption levels among high-income countries. This long-term decline has likely contributed to a markedly less cariogenic dietary environment for young children.

### Microbiological changes

Evidence suggests a reduction in *mutans streptococci* (MS) infection in Japanese preschool children. Seki and Yamashita^14^ reported that MS positivity among 3-year-olds declined from 68% to 38% between 1995 and 2000, paralleling decreases in ECC prevalence. This pattern strongly suggests that bacterial transmission and early infection dynamics – not improved enamel resistance to acid – played a primary role in the observed reductions in ECC.

### Maternal–child health system

Japan’s comprehensive MCH program includes repeated early-life contact, home guidance, anticipatory counselling, and early identification of at-risk families.^9^ Such longitudinal support may have amplified preventive behaviours independent of fluoride exposure.

### Social determinants

Improvements in education, reduced smoking prevalence, economic development, and better living environments likely contributed to broader enhancements in the conditions supporting child health, including oral health.^9^

### Limited effectiveness of 500 ppmF toothpaste

Evidence shows that 500 ppmF toothpaste provides only very modest caries-preventive effects in young children, consistent with WHO and FDI assessments that this concentration is insufficient for effective prevention.^6^^,^^11^ Small measurable effects have been reported in Japan, including modest associations between daily fluoride-toothpaste use and reduced risk of caries among 3-year-old children.^15^^,^^16^ However, these associations were weak and far too small to account for Japan’s substantial, decades-long national decline in ECC.

Other Japanese studies (not included here due to language restrictions) have reported minimal or no preventive benefit at this concentration. Moreover, any observed benefit may reflect use of toothpaste amounts exceeding both the recommended smear-size quantity and the ‘pea-size’ amount – levels at which 500 ppmF toothpaste is not expected to be effective – raising concerns about possible fluorosis risk if overused.

## Discussion

Japan’s four-decade decline in ECC provides a rare example of sustained improvement in early childhood oral health occurring largely in the absence of substantial fluoride exposure during infancy. The convergence of behavioural, dietary, microbiological, and social changes – supported by a comprehensive maternal–child health system – appears to have created an early-life environment increasingly protective against ECC. In addition, high participation rates may reflect a generally higher level of awareness, attitude, and behaviour towards better oral health in the population and may themselves contribute to improve oral health outcomes. These findings underscore the importance of considering population-level determinants when interpreting long-term disease trends.

In Japan, no nationally representative dental data exist for children aged 3 to 5 years, as population-based dental examinations are conducted at age 3 and then resume only at school entry. For this reason, the present analysis intentionally focuses on age 3 and does not attempt developmental comparisons beyond this point. This focus is deliberate. The period before age 3 represents a critical window for preventive decision-making, particularly regarding fluoride toothpaste use in children who cannot reliably expectorate. Recent empirical evidence from Europe highlights the practical difficulty of controlling fluoride toothpaste exposure in infancy. In a study of children up to 24 months, parents routinely dispensed amounts several-fold higher than recommended, and a substantial proportion of toothpaste was swallowed, underscoring the challenges of safe fluoride use before reliable expectoration is possible.^17^ The present findings therefore directly inform ongoing debates about the necessity and appropriateness of routine fluoride toothpaste use in infants and toddlers.

Several of the observed improvements preceded or occurred independently of changes in fluoride use. Reductions in sugar consumption, declining *mutans streptococci* infection, enhanced caregiver hygiene behaviours, and stable high participation in early-life dental checkups collectively created a preventive foundation strong enough to reduce ECC even under conditions of minimal fluoride exposure. These patterns highlight that effective early-life caries prevention is achievable through multiple pathways, particularly when upstream determinants improve simultaneously. Although direct national data on specific practices are limited, increased attendance in childcare and preschool settings, with structured daily routines and regulated snack times, may plausibly have contributed to reduced cariogenic exposure at the population level.

Japan’s experience does not diminish the importance of fluoride but clarifies its relative contribution in low-risk contexts. The long-term decline in ECC occurred while most infants used low-fluoride (500 ppmF) toothpaste and while water fluoridation was absent. This unique situation allows partial separation of fluoride effects from broader social and behavioural influences, offering insight into how non-fluoride determinants can sustain population-level improvements.

These findings also help contextualize ongoing debates surrounding the use of fluoride toothpaste in infants and toddlers. In settings where early-life caries risk is already low, the incremental benefit of routine fluoride toothpaste use before age two or three remains uncertain. Evidence gaps persist regarding both the effectiveness and the safe application of fluoride toothpaste in children who cannot rinse their mouths, and the dose required to achieve meaningful prevention is closely linked to the risk of fluorosis. A selective, risk-based approach – targeting infants with clearly elevated caries risk, while providing caregivers with balanced information about benefits and potential fluorosis risks – may therefore represent an appropriate strategy in low-risk countries such as Japan.

## Conclusion

Japan’s four-decade decline in ECC represents a rare natural social experiment in which substantial improvements occurred despite minimal fluoride exposure in infancy. The convergence of behavioural, dietary, microbiological, and social changes – supported by a comprehensive maternal–child health system – appears to have created an early-life environment increasingly protective against ECC, even in the absence of high fluoride availability. However, this study is purely descriptive and intended as a hypothesis-generating framework, and the relative contribution of the proposed non-fluoride factors cannot be inferred from the available data.

Japan’s experience does not diminish the value of fluoride but highlights the need to better understand the predominant non-fluoride factors operating during infancy. In addition, there remains an evidence gap regarding fluoride toothpaste use in infants – particularly the lack of demonstrated efficacy for smear-size applications and the fluorosis risk associated with doses shown to be effective. Providing caregivers with clear and balanced information about both potential benefits and fluorosis risks is essential to support genuinely informed decision-making.

Japan’s example illustrates the possibility that strengthening upstream determinants, coupled with thoughtful guidance on fluoride use, can shape sustainable and safe pathways for early-life caries prevention. Preventive strategies based on this concept could be adaptable to the diverse circumstances and preventive needs of countries around the world.

## CRediT authorship contribution statement

**Yoshihisa Yamashita:** Conceptualization, Data curation, Formal analysis, Investigation, Writing – original draft, Writing – review & editing.

## Conflict of interest

None disclosed.
